# Hexokinase II may be dispensable for CD4 T cell responses against a virus infection

**DOI:** 10.1371/journal.pone.0191533

**Published:** 2018-01-19

**Authors:** Siva Karthik Varanasi, Ujjaldeep Jaggi, Nissim Hay, Barry T. Rouse

**Affiliations:** 1 Department of Genome Science and Technology, University of Tennessee, Knoxville, Tennessee, United States of America; 2 Department of Biomedical and Diagnostic Sciences, College of Veterinary Medicine, University of Tennessee, Knoxville, Tennessee, United States of America; 3 Department of Biochemistry and Molecular Genetics, College of Medicine, University of Illinois at Chicago, Chicago, Illinois, United States of America; University of Georgia, UNITED STATES

## Abstract

Activation of CD4 T cells leads to their metabolic reprogramming which includes enhanced glycolysis, catalyzed through hexokinase enzymes. Studies in some systems indicate that the HK2 isoform is the most up regulated isoform in activated T cells and in this report the relevance of this finding is evaluated in an infectious disease model. Genetic ablation of HK2 was achieved in only T cells and the outcome was evaluated by measures of T cell function. Our results show that CD4 T cells from both HK2 depleted and WT animals displayed similar responses to in vitro stimulation and yielded similar levels of Th1, Treg or Th17 subsets when differentiated in vitro. A modest increase in the levels of proliferation was observed in CD4 T cells lacking HK2. Deletion of HK2 led to enhanced levels of HK1 indicative of a compensatory mechanism. Finally, CD4 T cell mediated immuno-inflammatory responses to a virus infection were similar between WT and HK2 KO animals. The observations that the expression of HK2 appears non-essential for CD4 T cell responses against virus infections is of interest since it suggests that targeting HK2 for cancer therapy may not have untoward effects on CD4 T cell mediated immune response against virus infections.

## Introduction

Recently it has become evident that cells of the immune system show distinct differences in the metabolic pathways they use [1,2]. This opens up the prospect of manipulating metabolism to shape the nature of immunity. A well-studied metabolic difference between cell types has been the glucose metabolic pathway by which T cells mainly derive their energy [3]. Thus, some subsets of T cells generate their ATP mainly by oxidative glycolysis, whereas others mainly use mitochondrial respiration [4]. With regard to oxidative glycolysis, the process is critically influenced by enzymes which include at least 4 hexokinase isoforms to generate glucose 6-phosphate from glucose (the first rate limiting step of glycolysis). Of the 4 isoforms, mainly two, HK1 and HK2, are expressed by T cells [5,6]. In addition, when T cells are activated, as occurs in some autoimmune diseases, the fold change in expression of HK2 far exceeds that of HK1 when compared to resting cells [6,7]. Moreover, HK2 has two tandem catalytically active domains whereas HK1 has only one catalytically active domain [8]. Taken together this could mean that HK2 may be more relevant than HK1 for T cell function, although this possibility has not been substantiated, particularly in vivo.

In an attempt to evaluate if HK2 is more relevant than HK1 in activated T cells, we bred appropriate mice strains that would delete HK2 specifically in T cells from the onset of the development. We could readily show that overall CD4 and CD8 T cell numbers were unaffected by HK2 deletion and that the function of CD4 T cells in vivo in a virus immunopathology model was basically unchanged. Nevertheless, some modest differences in responsiveness were shown in vitro such as proliferative responses to T cell receptor stimulation. However, overall the absence of HK2 had no major effect on CD4 T cell functions. Moreover, expression of HK1 was upregulated in the absence of HK2 which was likely compensating for HK2 deletion. The systemic deletion of HK2 in adult mice does not elicit adverse physiological consequences but inhibits tumor development in mouse models of cancers, where HK2 is highly expressed compared to normal cells [9]. The results presented here suggest that the systemic deletion of HK2 will not interfere with the immune response towards such tumor cells.

## Results and discussion

**Fig 1:**
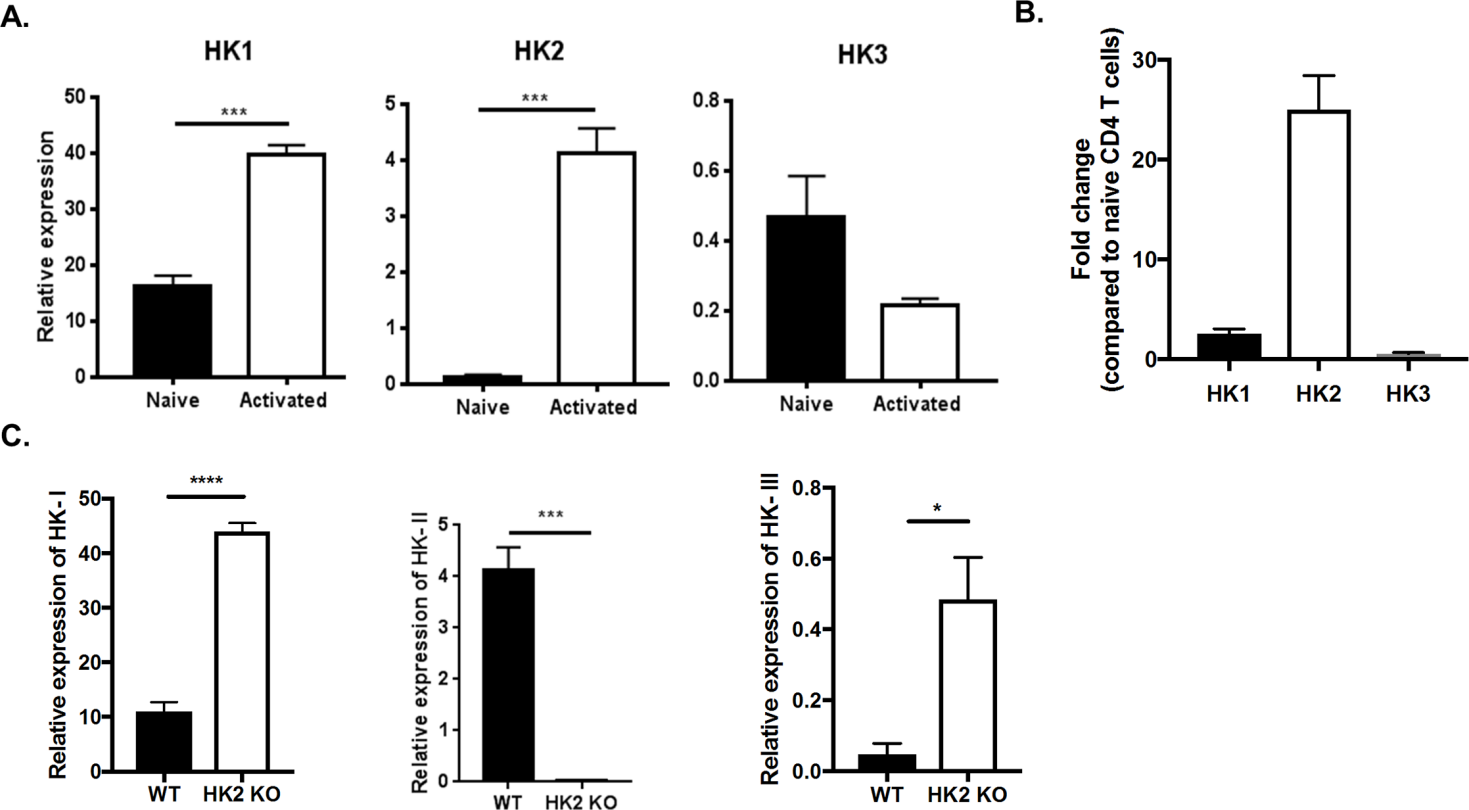
HK2 is up regulated upon CD4 T cell activation. (A) Naive CD4 T cells purified from C57BL/6 mice were cultured (100,000 cells/well) with 1μg/ml anti-CD3/CD28 for 24 hours followed by gene expression analysis by QRT-PCR compared to beta-actin. Bar graph representing expression of HK1, HK2 and HK3 in naïve and activated cells. (B) Bar graph of fold change in gene expression in activated cells compared to naïve cells (C) Naïve CD4 T cells were purified from WT and HK2 KO mice were activated anti-CD3/CD28 for 24 hours. Bar graph representing gene expression of HK, HK2 and HK3 compared to beta-actin. Data represents means ± SEM from two independent experiments (n = 3/group) P≤ 0.0001 (****), P≤0.001 (***), P≤0.05 (*).

As mentioned, previous studies showed that in activated T cells HK2 is up-regulated more than other hexokinases which could mean it is more relevant for T cell function. We confirmed this observation using real time PCR showing that upon TCR activation of CD4 T cells, the expression of HK2 was up-regulated 25–40 fold compared to naïve cells, whereas HK1 was up-regulated only about 3 fold (Fig 1B). However, the absolute expression level of HK1 in activated cells was still higher than HK2. The other isoforms HK3 and HK4 were barely detectable either in resting or activated T cells. Of note, resting T cells showed only minimal levels of HK2, whereas, the expression of HK1 was readily detectable (Fig 1A).

To ascertain if the dramatic up regulation of HK2 in activated T cells had physiological relevance compared to other hexokinases, mice were bred to delete the expression of the HK2 isoform specifically in T cells. The deletion was achieved by breeding CD4 Cre mice to HK2 flox/flox and homozygous pups (HK2 KO) were raised to maturity to evaluate and compare T cell responses to HK2 flox/flox animals (WT). The deletion of HK2 was confirmed by the absence of HK2 expression in enriched CD4 T cell populations after TCR stimulation in vitro as measured by RT-PCR (Fig 1C). Interestingly, deletion of HK2 also resulted in elevated levels of HK1 mRNA (~3 fold) in resting cells. Additionally, the expression of HK3 also increased although levels were still minimal (Fig 1C). These results were unexpected, since deletion of HK2 in several tumors did not result in elevated HK1 [9] indicating that T cells might depend less on HK2 and have compensatory mechanisms distinct from cancer cells.

**Fig 2:**
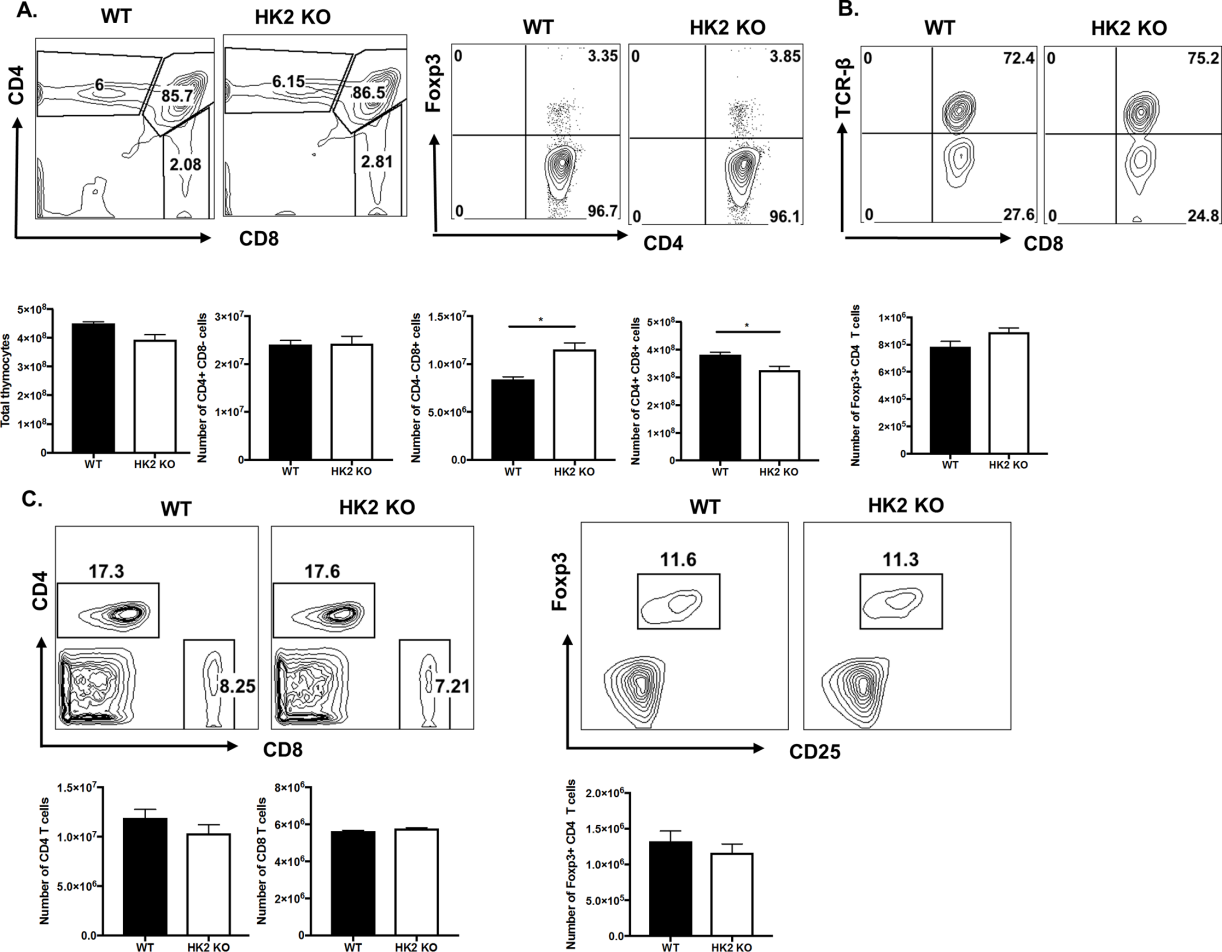
T cell specific HK2 deletion is dispensable for T cell development. Thymus and spleens from 5–6 week old naïve WT and HK2 KO animals were isolated. (A) Representative FACS plots and bar graph showing the frequency and number of total thymocytes, CD4+ CD8- T cells, CD4- CD8+ T cells, CD4+CD8+ T cells and CD4+CD8- Foxp3+ Treg cells in Thymus. (B) Representative FACS plot showing the TCR-beta expression gated on CD8+ CD4- T cells in the thymus. (C) Representative FACS plots and histogram showing the frequency and number of CD4+ CD8- T cells, CD4- CD8+ T cells and CD4+CD8- Foxp3+ Treg cells in Spleen. Gated on live cells. Data represents the mean ± SEM of more than 2 independent experiments (n = 3 mice/group). P≤0.001(*).

Experiments were also done to measure the impact of HK2 deletion in T cell development and function. HK2 deletion showed no major effect on CD4 T cell development, as the number of single positive (SP) CD4 and Treg in the HK2 KO and WT mice in both the thymus and spleen showed no statistically significant differences (Fig 2A and 2C). The number of SP CD8 T cells was only minimally increased in the thymus and not in the spleen. However, no significant difference in expression of TCR beta on SP CD8 T cell thymocytes was observed (Fig 2B). Hence, HK2 might be dispensable for CD4 T cell development. It remains to be known why HK2 deletion has resulted in minimal changes in CD8 T cell numbers in the thymus. Curiously the deletion of HK2 in some other tissue does have consequences to their development. These tissues include heart, muscle and adipose tissue [10,11].

**Fig 3:**
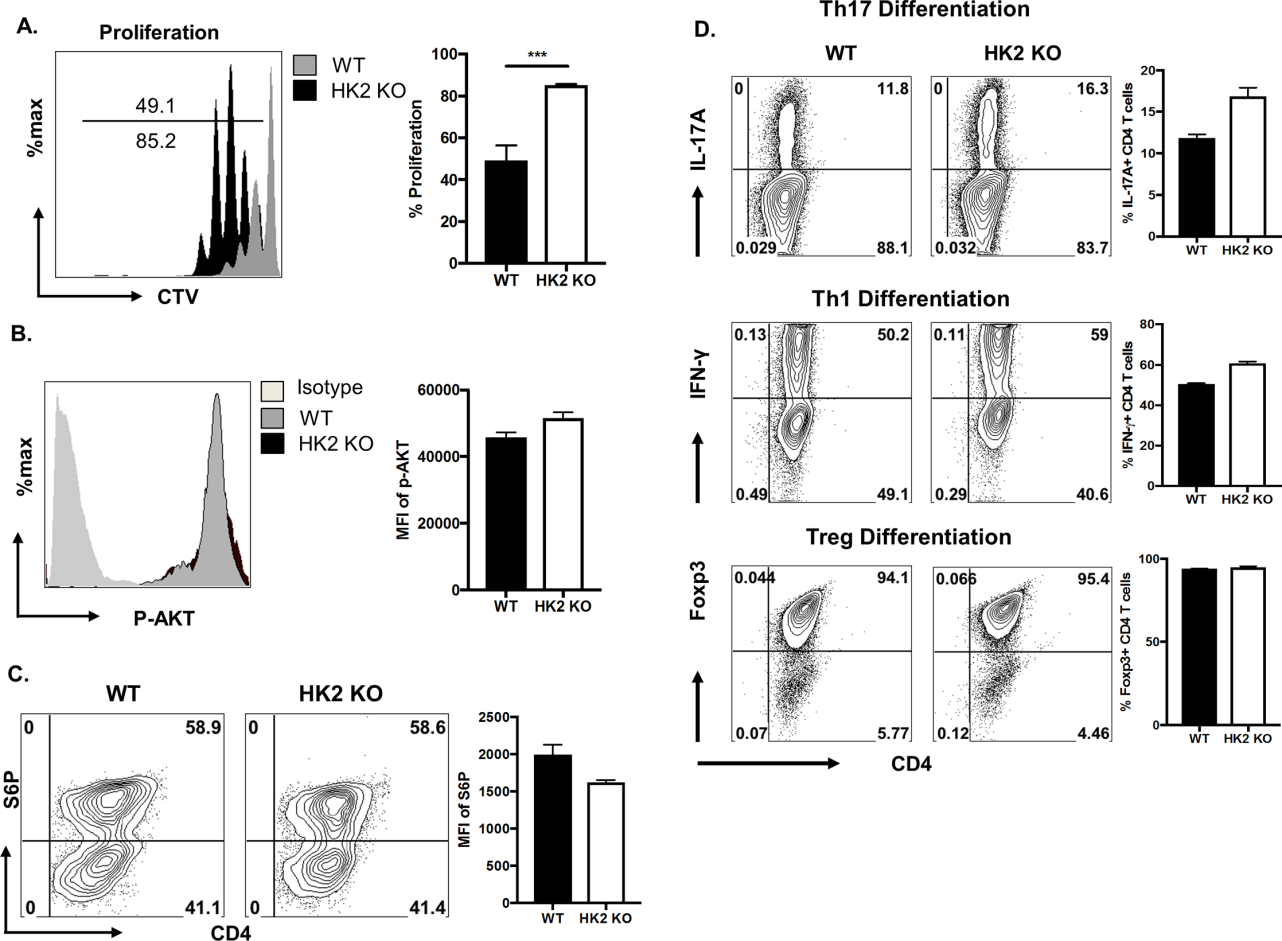
HK2 deletion led to enhanced CD4 T cell proliferation without affecting T cell differentiation in vitro. (A-C) Naïve CD4 T cells from WT and HK2 KO mice were CTV labelled and activated for 72 hours. (A) Representative FACS plots and bar graph indicating CTV dilution (as a measure of proliferation) gated on live CD4 T cells. Representative FACS plots and Histogram representing the levels of (B) phosphorylated–AKT and (C) Phosphorylated-S6 kinase. (D) Naïve CD4 T cells from WT and HK2 KO mice were cultured in the presence Treg or Th1 or Th17 differentiating conditions for 5 days followed by re-stimulation for 4 hours with PMA/Ionomycin. Histogram showing frequency of Th17, Th1 cells and Treg cells. All the measurements were made on live CD4 T cells. Data represents means ± SEM from two independent experiments (n  =  3/group) P≤0.001(***).

Additional experiments were done to measure the functional and metabolic consequences of HK2 deletion. Isolated CD4 T cells from KO and WT were TCR stimulated in vitro and responses were compared. Some modest differences were observed. Thus KO CD4 T cells generated approximately 1.5 fold greater proliferative responses to TCR stimulation compared to WT T cells (Fig 3A). However, the response to activation measured by induction of phosphorylated AKT and the phosphorylation of S6 kinase (indicative of mTOR activity) revealed no significant differences between WT and KO cells (Fig 3B and 3C). In addition, no significant differences were observed when cells from KO and WT were differentiated in vitro into Th1, Th17 and Treg populations (Fig 3D).

**Fig 4:**
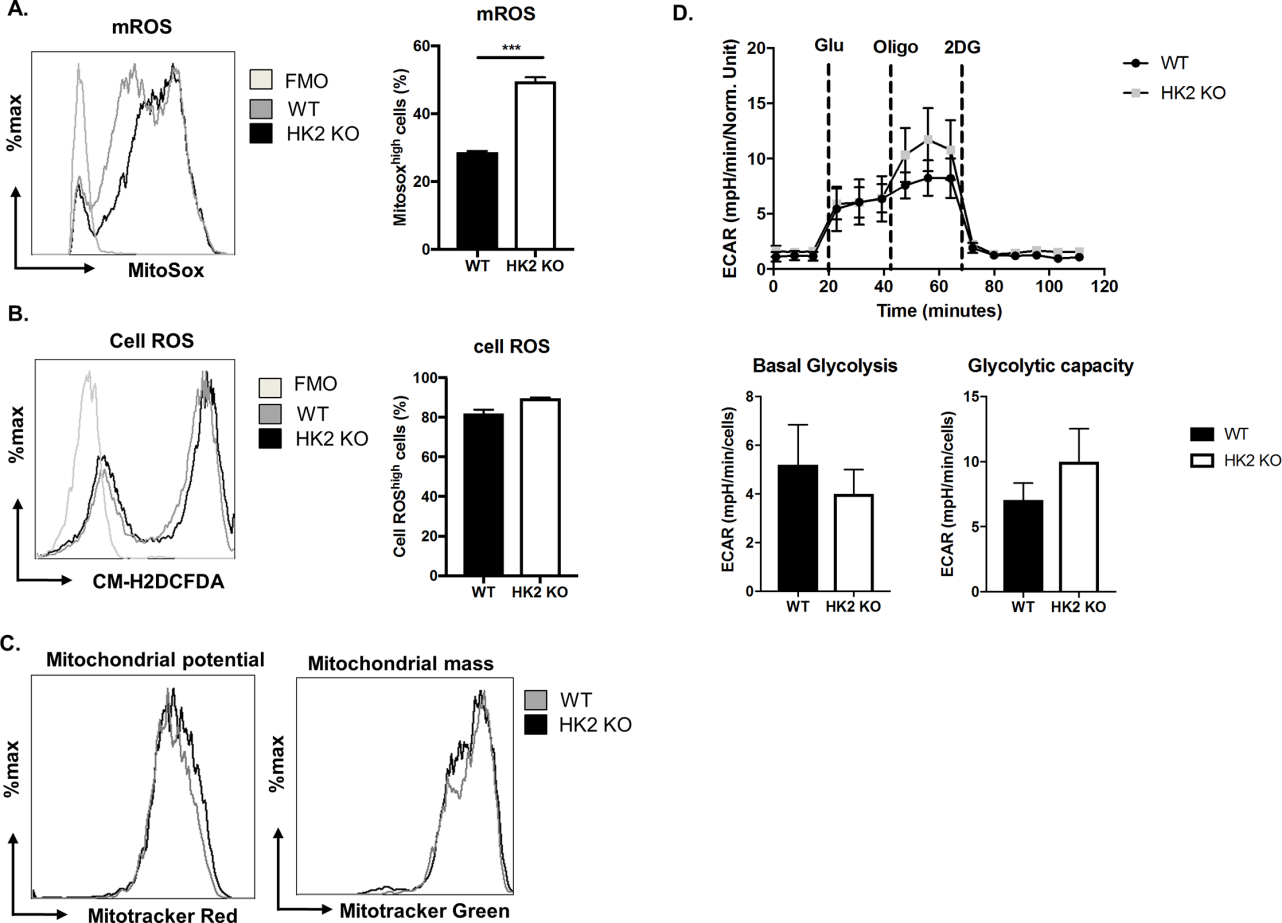
HK2 deletion in T cells had minimal effect on T cell glycolysis. (A-C) Naïve CD4 T cells from WT and HK2 KO mice were activated for 72 hours. (A) mROS (MitoSOX high) producing cells, (B) Cellular ROS (CM-H2DCFDA) producing cells and (C) Mitochondrial membrane potential (MitoTracker Red CM-H2Xros) and mitochondrial mass (MitoTracker Green). Data represents means ± SEM from three independent experiments (n  =  3/group). FMO, fluorescence minus one. (D) Naïve CD4 T cells were purified from WT and HK2 KO mice and cultured with 1μg/ml anti-CD3/CD28 for 72 hours. Line graph showing changes in Extracellular acidification rates (ECAR) following addition of glucose, oligomycin and 2DG and bar graph showing basal glycolysis, glycolytic capacity. n = 6-8/group.

The explanation for the higher proliferative response in KO CD4 T cells was not resolved but it could relate to suppressive effects that HK2 may have on mitochondrial function. Thus, activated T cells from HK2 KO animals displayed around 2-fold higher mitochondrial ROS (mROS) production without affecting cellular ROS levels (Fig 4A and 4B). Also, the increased mitochondrial ROS is associated with only moderate increase in mitochondrial membrane potential as measured my MitoTracker Red CM-H2Xros with little or no change in mitochondrial mass as measured by MitoTracker green (Fig 4C). This is in line with the observation in some cell lines and neurons that HK2 binds to mitochondria via voltage-dependent anion channels and control mitochondrial function by inhibiting mROS generation [12,13]. Moreover, T cells that lack the ability to generate mROS display reduced proliferation and activation [14]. In conclusion, deletion of HK2 induced mROS which might have led to a modest increase in the proliferation of CD4 T cells. Since intact mitochondrial function is critical for effective memory T cell responses [15], the effects of HK2 deletion on established memory T cells needs to be evaluated using an inducible Cre system in adult mice with existing memory responses.

With regard to metabolism, activated CD4 T cells from WT and HK2 KO mice showed similar levels of glycolysis and glycolytic capacity as measured by extracellular flux analysis (Fig 4D). These data likely mean that the absence of HK2 was being compensated by other hexokinase isoforms. This finding was in contrast to the findings in some cancer cells where, deletion of HK2 did result in reduced proliferation and glycolysis [9,16]. Although, the mechanistic reasons for such disparities were not evaluated in this report, we speculate that HK1, whose levels were elevated in the absence of HK2 could compensate for the function that HK2 was performing in activating CD4 T cells.

**Fig 5:**
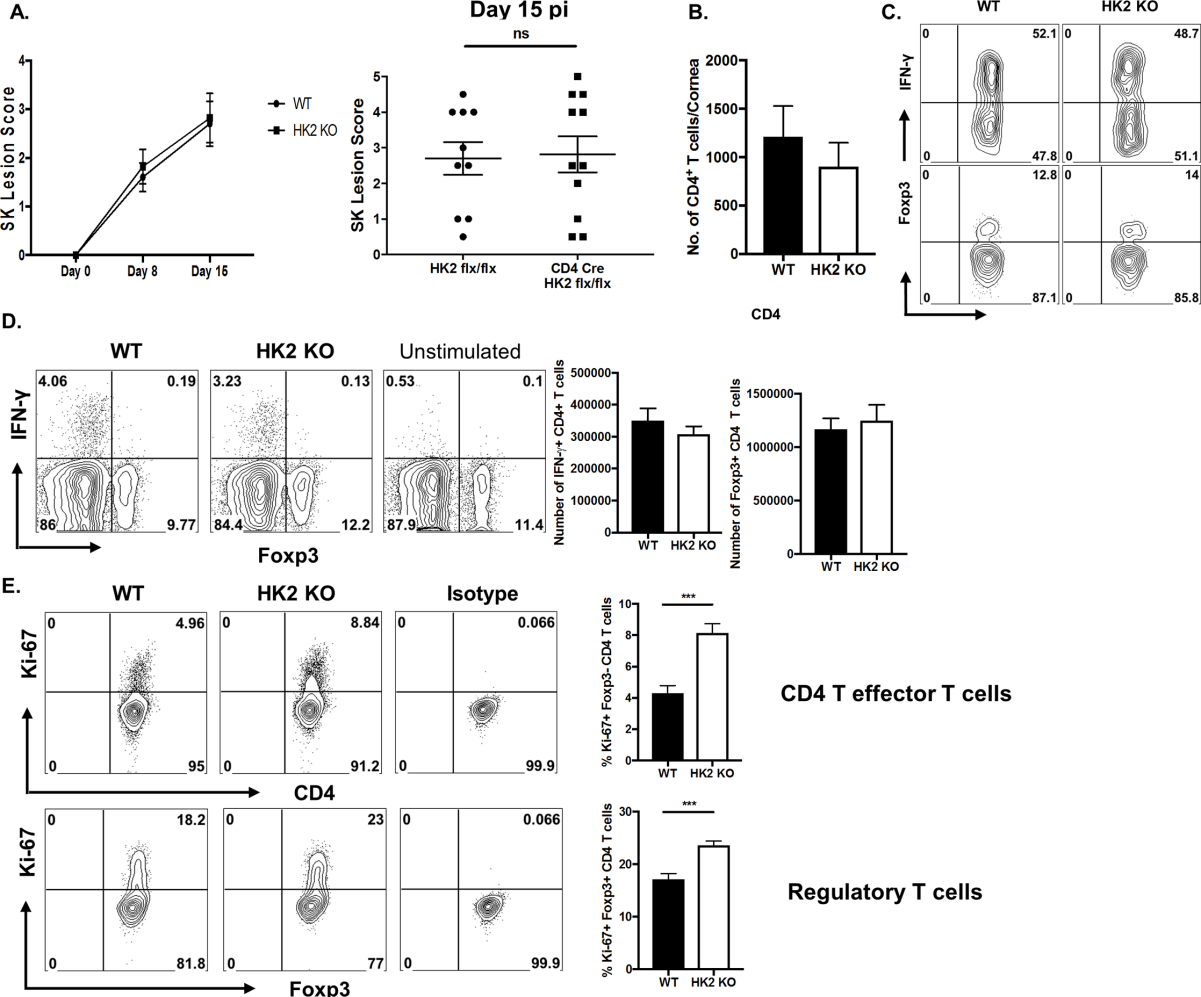
HK2 deletion displayed minimal effects on CD4 T cell responses but higher proliferation upon ocular infection with HSV-1. WT and HK2 KO animals were infected with 1 x 10^4^ PFU of HSV-RE. (A) Individual eye scores of stromal keratitis lesion severity on day 15 pi. (B) Representative histogram showing the number of total CD4+ T cells infiltrating the cornea at day 15 pi (C) Pool of corneas were stimulated with PMA/Ionomycin, representative histogram showing the number of Th1 (Live CD4+ IFN-γ) cells and Treg (Live CD4+ Foxp3) cells in the cornea at day 15 pi. (D) DLN were stimulated with PMA/Ionomycin, representative FACS plots and histogram showing frequency and number of Th1 (live CD4+ IFN-γ^pos^) and Treg (live CD4+ Foxp3^pos^). Gated based on the Unstimulated control. (E) Representative FACS plots and histogram showing frequency of proliferating (Ki-67^pos^) effector (Live CD4+ Foxp3^neg^) and regulatory T cells (Live CD4+ Foxp3^pos^). Gated based on the Isotype. Data represents the mean ± SEM of more than 3 independent experiments (n = 3–10 mice/group). P≤0.05(*).

**Fig 6:**
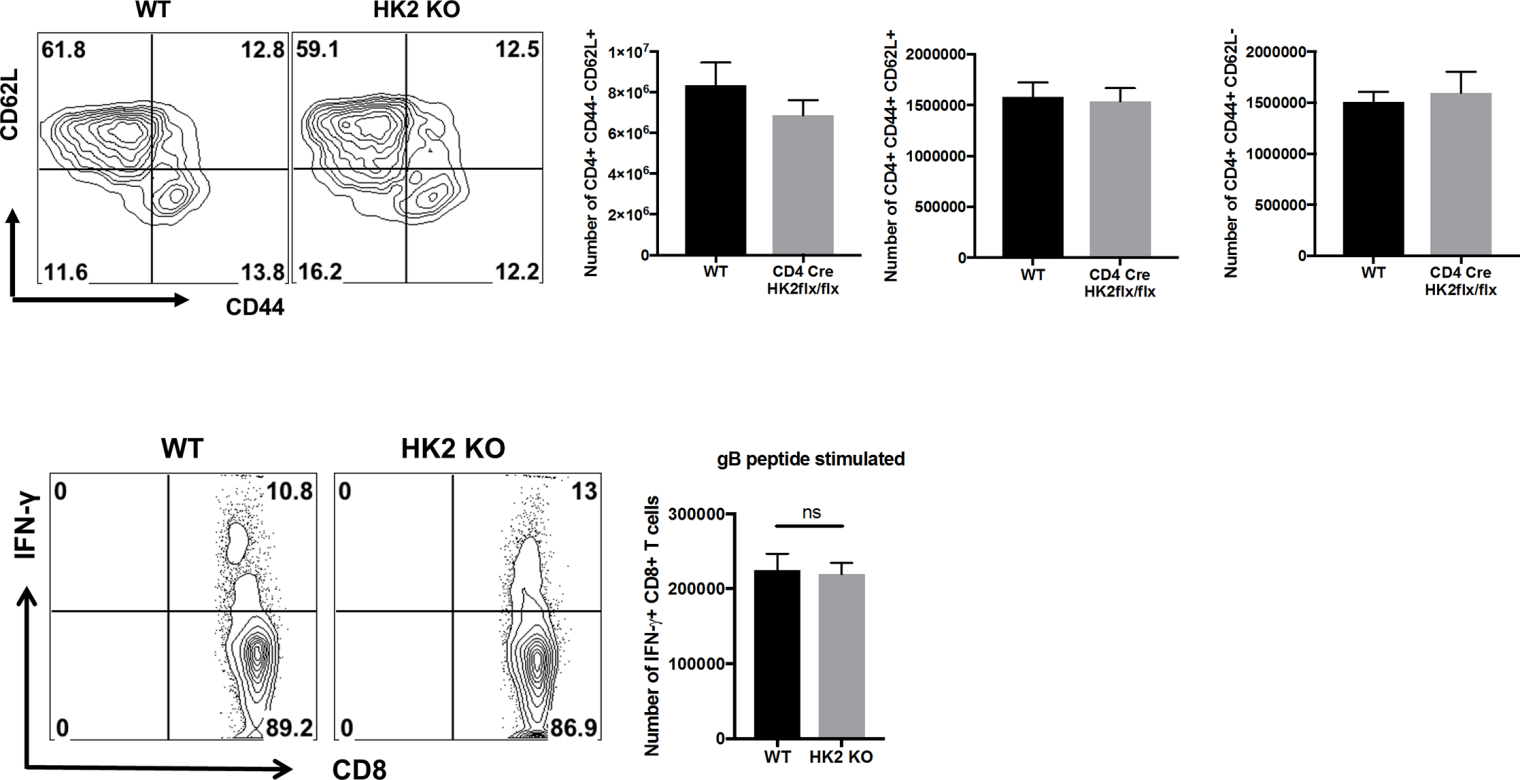
HK2 deletion in T cells had minimal effects on HSV-1 specific CD8 T cells and expression of CD44 and CD62L on CD4 T cells. (A) WT and HK2 KO animals were ocularly infected with HSV-1 and day 15pi draining cervical lymph nodes were isolated. Representative FACS plots and bar graphs showing the frequency and number of activated (CD44+CD62L-), naïve (CD44-CD62L+) and memory CD4 T cells (CD44+CD62L+). Data represents means ± SEM from three independent experiments (n = 3/group). (B) WT and HK2 KO animals were infected in their footpad with HSV-1 KOS and at day 4pi popliteal lymph nodes were isolated. Representative FACS plots and bar graph indicates the frequency and number of CD8 T cells expressing IFN-gamma upon stimulation with gB peptide. Data represents means ± SEM from two independent experiments (n = 3/group).

Finally, the effects of HK2 deletion on the outcome of CD4 T cell function in vivo were assessed. Age and sex matched WT and HK2 KO animals were ocularly infected with HSV-1 and the severity of lesions of stromal keratitis were compared. The results revealed no significant differences in responses in the two groups. Thus lesions were of comparable severity and the number of T cells present in lesions was basically the same including the proportion of infiltrating Th1 and Treg. (Fig 5A–5C). Similar to the in vitro data, the number and frequency of Treg and IFN-γ producing CD4 T cells in the DLN were not significantly different, despite some increased proliferation of both of CD4 T cell subsets (effectors and regulators) as measured by Ki-67 staining (Fig 5D and 5E). Conceivably, the disparity between increased proliferation, yet similar inflammatory reactions could mean that some of the proliferating T cells of HK2 KO animals were undergoing apoptosis, an issue that is being further evaluated. Of note, the frequency and the number of CD44 and CD62L expressing CD4 T cells in the DLN at day 15 pi remained unchanged (Fig 6A). To measure if HK2 deletion had an effect on HSV-1 specific CD8 T cell responses (a CD4 helper cell dependent response)[17], a well-established footpad immunization model was used [18]. Cell suspensions of draining lymph nodes were stimulated with a gB peptide, which is the immuno-dominant peptide recognized by B6 mice [19]. The results, measured by the ICS assay for HSV specific IFN-gamma producing CD8 T cells, revealed no significant differences in responses by WT and HK2 KO animals (Fig 6B). In addition since HK2 deletion was done using CD4 Cre mice, CD8 T cells and some subsets of APC would also be deficient of HK2. However, such potential effect did not affect the outcome of a virus specific CD8 T cell response.

## Conclusions

Our finding that HK2 function can be dispensed with for CD4 T cell function could come as welcome news to the cancer therapy field. Thus for some cancers targeting HK2 with inhibitory drugs is an objective [20]. Our results would argue that such therapy may not be accompanied by negative effects on CD4 T cell functions which are necessary for anti-microbial protection and in some cases for anti-tumor effects as well.

## Materials and methods

### Ethics statement

This study was carried out in strict accordance with the recommendations in the Guide for the Care and Use of Laboratory Animals of the National Institutes of Health and guidelines of the Institutional Animal Care and Use Committee, and adhered to the ARVO Statement for the Use of Animals in Ophthalmic and Vision Research. The protocol was approved by the University of Tennessee Animal Care and Use committee (IACUC) (protocol approval numbers 1244). All procedures were performed under Tribromoethanol (Avertin) anesthesia, and all efforts were made to minimize suffering.

### Mice and virus

Female C57BL/6 mice were purchased from Harlan Sprague-Dawley, Inc. (Indianapolis, IN), CD4 Cre mice (C57BL/6 background) mice were purchased from Jackson laboratories and HK2 flox/flox (C57BL/6 background) were a kind gift from Dr. Nissim Hay (University of Illinois, Chicago)[9]. CD4 Cre mice were bred to HK2 flox/flox mice and the pups that were positive for Cre and homozygous for HK2flox/flox (confirmed by genotyping) were used as HK2 KO and mice that were homozygous for only HK2flox/flox and negative for Cre were used as WT controls for all the experiments. HSV-l RE strain was used in all procedures.

### HSV-1 ocular infection and clinical scoring

Corneal infections of 5–6 week old mice were conducted as previously described [21]. Briefly, mice were anesthetized by intra peritoneal (i.p) injection of Tribromoethanol (Avertin). Mice were scarified on cornea with a 27-gauge needle, and a 3 μl drop containing 1x10^4^ PFU of HSV in 3μl volume was applied to the eye. The eyes were examined on different days post infection (dpi) with a slit-lamp biomicroscope (Kowa Company, Nagoya, Japan), and the clinical severity of keratitis of individually scored mice was recorded as previously described [21].

### Footpad infection with HSV-1

Footpad infections on WT and HK2 KO (5–6 week old) animals were done as previously described [22]. Briefly, mice were deep anesthetized as described above and 30μl of 4x10^5^ PFU HSV-1 KOS was subcutaneously injected in each hind footpad. Mice were sacrificed 4 days post infection and the draining popliteal lymph nodes for isolated for ICS assay.

### Flow cytometric analysis

The single-cell suspensions obtained from corneal samples, draining cervical lymph nodes, Thymus and Spleen were stained for different cell surface molecules for fluorescence-activated cell sorting (FACS) analyses as described previously [23]. Proliferation assays were performed using Cell Trace Violet (CTV) labelled naïve CD4 T cells (2 X 10^5^ cells/well) from WT and HK2 KO stimulated with plate bound anti-CD3/CD28 Ab (1 μg/ml or 5 μg/ml) for 3 days. Naïve CD4 T cells (2 X 10^5^ cells/well) were stimulated for 72 hours in the presence of IL-2 and later labelled with MitoSOX (5μM), CM-H2DCFDA (1.25μM), MitoTracker Red CM-H2Xros (100nM) or MitoTracker green (100nM) and incubated for 30 min followed by live/dead staining and cytometric measurement. For HSV-1 specific CD8 T cells responses, 1x10^6^ single cell suspensions popliteal lymph node were stimulated in a 96 well U-bottom plate. Cell were either left unstimulated or stimulated with SSIEFARL peptide (1μg/ml), for 5 h at 37°C in 5% CO2 in the presence of Brefeldin A (10μg/ml) followed by ICS assay.

### Reagents and antibodies

CD4 (RM4-5), IFN-γ (XMG1.2), CD25 (PC61, 7D4), CD44 (IM7), Annexin-V, Foxp3 (FJK-16S), anti-CD3 (145-2C11), anti-CD28 (37.51), GolgiPlug (Brefeldin A) from either ebiosciences or BD biosciences. P-AKT (S473-SDRNR) from ebiosciences and P-S6 (S235/236-D57.2.2E) from cell signaling. Phorbol 12-myristate 13-acetate (PMA) and Ionomycin from sigma. Cell Trace Violet, Live/Dead Fixable Violet Dead Cell Stain Kit, MitoTracker Red CM-H2Xros, MitoTracker Green, MitoSOX and CM-H2DCFDA from Life Technologies. Recombinant IL-2, IL-12, IL-6 and TGF-β from R&D systems. Glucose free RPMI media (life technologies) was prepared using dialyzed FBS. HSV-1 gB498–505 peptide (SSIEFARL) was from Genscript.

### Quantitative PCR (qPCR)

Taqman gene expression assays for HK1 (Hexokinase 1), HK2 (Hexokinase 2), HK3 (Hexokinase 3) and HK4 (Hexokinase 4) from Applied Biosystems were performed on using 7500 Fast Real-Time PCR system (Applied Biosystems) as described previously (18).

### Purification of CD4+ T cells

Naïve CD4+ T cells were purified using a mouse naïve CD4+ T cell isolation kit (Miltenyi Biotec, Auburn, CA). The purity was achieved at least to an extent of 90%.

### In vitro Treg, Th17 and Th1 differentiation assays

Naïve CD4 T cells were isolated from splenocytes of WT and HK2 KO mice as described above. Th1, Th17 and Treg cells were differentiated as described previously with some modifications [21,24]. Briefly, 1×10^6^ cells were cultured with plate bound anti-CD3/CD28 Ab (1 μg/ml) containing either Treg differentiating conditions: rIL-2 (100 U/ml) and TGFβ (5ng/ml) or Th1 differentiating conditions: IL-12 (5ng/ml) and anti-IL-4 (10 μg/ml) or Th17 differentiating conditions: IL-6 (25ng/ml) and TGFβ (5ng/ml) with anti-IL-4 (10 μg/ml) and anti-IFN-γ (10 μg/ml) for 5 days at 37°C in a 5% CO2 incubator. After 5 days, samples were re-stimulated with PMA and Ionomycin to measure Foxp3 expressing, IFN-γ and IL-17A producing CD4 T cells using flow cytometry.

### OCR and ECAR measurement

Naïve CD4 T cells from WT and HK2 KO were activated for 3 days in the presence of anti-CD3/CD28 (1 μg/ml). 1×10^6^ cells per well were plated on XF24 plate (Seahorse Bioscience). ECAR values were measured for glycolysis stress test using a Seahorse XF24 metabolic analyzer as previously described [23].

### Statistical analysis

Statistical significance was determined by Student's t-test. A P-value of <0.05 was regarded as a significant difference between groups: *P ≤ 0.05, **P ≤ 0.01, ***P ≤ 0.001. GraphPad Prism software (GraphPad Software, Inc., La Jolla, CA) was used for statistical analysis.

## Abbreviations

HSV-1: herpes simplex virus-1
Treg: regulatory T cell
HK: hexokinase
WT: wild type
